# Which Target Temperature for Post-Anoxic Brain Injury? A Systematic Review from “Real Life” Studies

**DOI:** 10.3390/brainsci11020186

**Published:** 2021-02-03

**Authors:** Andrea Minini, Filippo Annoni, Lorenzo Peluso, Elisa Gouvêa Bogossian, Jacques Creteur, Fabio Silvio Taccone

**Affiliations:** Department of Intensive Care, Erasmus Hospital, Université Libre de Bruxelles (ULB), Route de Lennik, 808, 1070 Brussels, Belgium; dott.minini.andrea@gmail.com (A.M.); filippo.annoni@erasme.ulb.ac.be (F.A.); lorenzopeluso80@gmail.com (L.P.); elisagobog@gmail.com (E.G.B.); jcreteur@ulb.ac.be (J.C.)

**Keywords:** targeted temperature management, dose, cardiac arrest, 33 °C, 36 °C, outcome

## Abstract

There is a persistent debate on the optimal target temperature to use during cooling procedures in cardiac arrest survivors. A large randomized clinical trial (RCT) including more than 900 patients showed that targeted temperature management (TTM) at 33 °C had similar mortality and unfavorable neurological outcome (UO) rates as TTM at 36 °C in out-of-hospital cardiac arrest patients with any initial rhythm. Since then, several observational studies have been published on the effects of changes in target temperature (i.e., from 33 to 36 °C) on patients’ outcome. We performed a systematic literature search from 1 January 2014 to 4 December 2020 and identified ten retrospective studies (very low levels of certainty; high risk of bias), including 5509 patients, that evaluated TTM at 33 °C vs. TTM at 36 °C on the occurrence of UO (n = eight studies) and mortality (n = ten studies). TTM at 33 °C was associated with a lower risk of UO when studies assessing neurological outcome with the Cerebral Performance Categories were analyzed (OR 0.80 [95% CIs 0.72–0.98]; *p* = 0.03). No differences in mortality were observed within the two TTM strategies. These results suggest that an inappropriate translation of TTM protocols from large well-conducted randomized trials into clinical management may result in unexpected effects on patients’ outcome. As for all newly commercialized drugs, epidemiological studies and surveillance programs with an adequate follow-up on large databases are necessary to understand how RCTs are implemented into medical practice.

## 1. Introduction

The most severe complication of sudden cardiac death is hypoxic-ischemic brain injury (HIBI) [1]; most patients admitted to an intensive care unit (ICU) after resuscitation from either out-of-hospital (OHCA) or in-hospital cardiac arrest (IHCA) are comatose and about two-thirds of them will eventually die before hospital discharge because of irreversible brain damage [2]. Moreover, severe cognitive dysfunction can affect up to 20% of cardiac arrest survivors with favorable neurological recovery [3] and potential neuroprotective therapies remains one of the most relevant strategies in this setting [4].

The only therapeutic intervention that has shown some benefits to mitigate the sequelae of HIBI is the use of targeted temperature management (TTM); at this moment, three randomized clinical trials (RCTs) have demonstrated a significant improvement in the proportion of patients with favorable neurological outcome when cooling at 33 °C for 24 h was compared to no temperature control or normothermia [5,6,7]. Nevertheless, although TTM remains still recommended into international guidelines [8], the level of evidence supporting its use remains low (because of several methodological bias present into initial trials [5,6]), many concerns on clinically relevant effectiveness have been raised also for experimental studies [9] and a large trial has been recently completed (NCT03114033) and will eventually provide a definitive answer on the role of TTM in this setting.

In clinical practice, one of the most controversial issues remains the target temperature to use during the cooling phase; in 2013, a large RCT including more than 900 patients compared TTM at 33 °C to 36 °C in OHCA patients with any initial rhythm and reported similar mortality and favorable neurological outcome rate in the two groups [10]. If, on one side, some authors have claimed that TTM at 36 °C should become the standard strategy for all cardiac arrest patients [11], others have highlighted a decreased utilization of TTM after the publication of this trial (as 36 °C was interpreted as being equivalent to normothermia) or reported a reduction of strict temperature control in these patients, with a potential increased risk of poor neurological outcome [12,13]. As such, despite guidelines suggested to implement TTM in cardiac arrest survivors using a target temperature between 32 and 36 °C [8], the decision to target 36 °C in clinical practice may have led to a less rigid adherence to TTM, including high temperature variability, less use of sedation to avoid shivering and a higher occurrence of fever. Data from national registries reported a progressive decrease in the use of TTM after the trial published in 2013, which was associated with a reduced patients’ survival over time [14]. To provide more information on the optimal target temperature during TTM, we therefore, analyzed the existing literature reporting data from different studies evaluating changes in practice (i.e., from 33 °C to 36 °C) and tried to summarize the overall effects on patients’ mortality and neurological outcome.

## 2. Materials and Methods

### 2.1. Search Strategy and Study Selection

We adhered to the Preferred Reporting Items for Systematic Reviews and Meta-Analysis-Protocols (PRISMA-P) guidelines [15].

### 2.2. Data Sources and Search Strategies

A systematic literature search from 1 January 2014 to 4 December 2020 was performed on PubMed using the following terms: (“targeted temperature management” OR “TTM” OR “hypothermia” OR “cooling” OR “therapeutic hypothermia” OR “33 C” OR “36 C”) AND (“cardiac arrest” OR “heart arrest” OR “post anoxic brain injury” OR “hypoxic ischemic encephalopathy”). All observational studies published in English in peer-reviewed journals and comparing the use of TTM at 33 vs. 36 °C in adults (>18 years of age) were eligible for inclusion.

The research question was: (1) In cardiac arrest survivors (participants), is TTM at 33 °C (intervention), when compared to TTM at 36 °C (control), associated with similar neurological outcome and mortality (outcome) in observational studies (study) published after the “TTM trial” [10]? We also reviewed reference lists from original manuscripts and published systematic reviews and meta-analyses to identify studies that were not listed in the original database search.

### 2.3. Study Screening and Selection

Two authors (AM and FA) independently screened study titles and abstracts for potential eligibility and assessed their validity. Disagreement between authors was assessed and resolved through a third reviewer (FST), who reviewed the original text of the article. None of the authors of the original studies was contacted to obtain further information, which were not available in the published manuscript. All *post hoc* analyses from the “TTM trial” [10] were excluded. Additionally, an additional RCT reporting data from different target temperature was excluded [16]. Editorials, commentaries, letters to editor, opinion articles, reviews, meeting abstracts, case reports, and studies published in other languages were also excluded, as well as original articles lacking abstract and/or quantitative details on neurological outcome and survival. Only studies that met all the above criteria were incorporated for quantitative synthesis.

### 2.4. Appraisal of Study Quality

The level of evidence (LOE) of each study was assessed according to the Grading of Recommendations, Assessment, Development and Evaluations (GRADE) evidence system [17]. The risk of bias (ROB) for all these non-randomized studies was assessed using the ROBINS-I tool from the Cochrane database [18]. LOE was further analyzed by two experts (FST, FA) and one independent statistician. Disagreement was resolved by consensus.

The primary outcome of the meta-analysis was the occurrence of unfavorable neurological outcome (UO), whenever it was recorded. UO was defined according to the definition of each study; however, as UO is generally identified as a Cerebral Performance Category (CPC) of 3–5 in most of studies conducted in the cardiac arrest field, a subgroup analysis including only studies reporting CPC 3–5 as. UO was performed. Secondary outcome was mortality, whenever it was recorded; in case of several time-points of mortality assessment, the longest delay from arrest was considered. Subgroups analyses were performed for: (a) Different timing of outcome assessment (i.e., intensive care unit, ICU, discharge; hospital discharge; 30 days; 6 months); (b) only OHCA patients; and (c) only patients presenting with an initial shockable rhythm.

### 2.5. Statistical Analyses

Statistical analysis was conducted by Review Manager 5.3 software. Means of UO and mortality probabilities were obtained by weighting each study by the inverse of variance. The Mantel–Haenszel method was chosen as the reference method for fixed effects analysis. A Z test was carried out to assess the significance of the risk differences. Odds ratio (OR) and 95% confidence intervals (CIs) for UO and mortality were calculated with the Wilson method and placed in forest plots and statistical significance was assumed for *p* < 0.05. The I^2^ was calculated by χ^2^ test to assess variability due to heterogeneity rather than chance. A substantial heterogeneity was assumed with I^2^ > 50%. No funnel plot was performed as the total number of selected studies was less than 10.

## 3. Results

**Figure 1:**
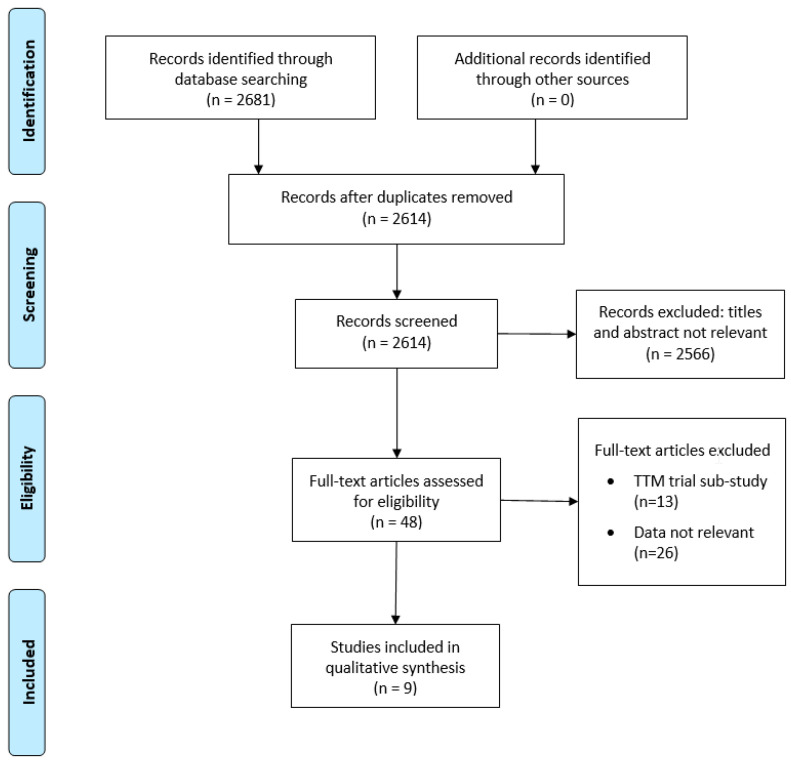
Flow diagram of the search results and selection of eligible studies. TTM = target temperature management.

A total of 2681 records were identified after the initial search. After the first screening procedure, 48 studies were assessed for eligibility. Of those, 39 were excluded and ten retrospective studies [12,13,19,20,21,22,23,24,25], including 5509 patients, were; therefore, included for the meta-analysis (Figure 1).

The characteristics of the selected studies are summarized in Table 1; all studies included OHCA patients; three studies also reported outcome for IHCA patients [20,21,22], and one study specifically included traumatic and post-operative cardiac arrest [21]. The ROB for all studies was high. The LOE was classified as very low.

### Unfavorable Neurological Outcome and Mortality

Unfavorable neurological outcome (n = 3155) was defined in six studies as Cerebral Performance Category (CPC) of 3–5 [13,22,23,24,25], as CPC 3–5 and 4–5 in one study [20] and as the inability to follow commands in another [21]. Neurological assessment was recorded either at hospital discharge (n = 6), at 30 days (n = 1) or at six months after arrest (n = 1).

**Figure 2:**
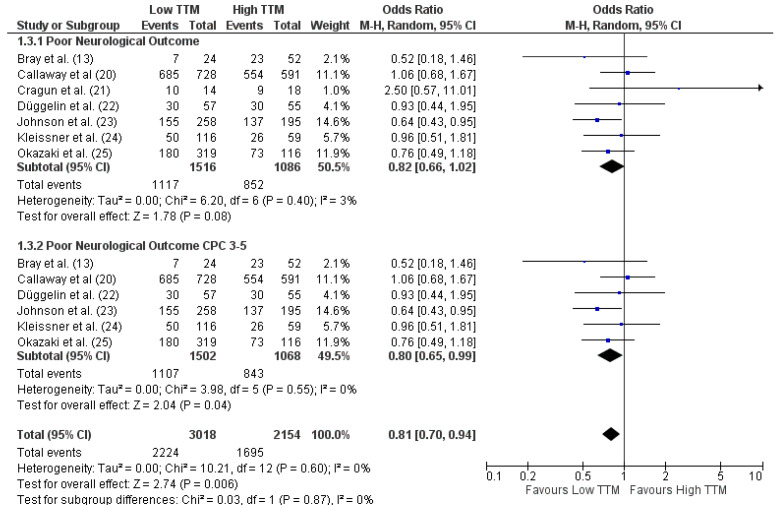
Forest plot for unfavorable neurological outcome in all patients with reported outcome and in all those with reported CPC 3–5. Low TTM = TTM at 33 °C; High TTM = TTM at 36 °C. The size of the squares for the risk ratio reflects the weight of the trial in the pooled analysis. The horizontal bars represent 95% confidence intervals (CIs) [13,20,21,22,23,24,25].

Overall, patients treated with TTM at 33 °C showed similar UO than those treated at 36 °C (OR 0.85 [95% CIs 0.73–0.99], *p* = 0.04—Figure 2). However, when only studies reporting UO as CPC 3–5 were considered (n = 4280), patients treated with TTM at 33 °C showed a lower probability of UO than those treated at 36 °C (OR 0.84 [95% CIs 0.72–0.98], *p* = 0.03) than those treated at 36 °C (Figure 2). No significant heterogeneity was observed among these studies for UO assessment (I^2^ of 0%).

**Figure 3:**
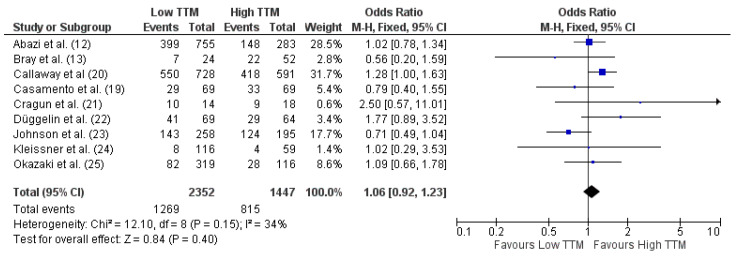
Forest plot for mortality in all patients with reported outcome. Low TTM = TTM at 33 °C; High TTM = TTM at 36 °C. The size of the squares for the risk ratio reflects the weight of the trial in the pooled analysis. The horizontal bars represent 95% confidence intervals (CIs) [12,13,19,20,21,22,23,24,25].

Mortality was reported in all studies, either at hospital discharge (n = 7), ICU discharge (n = 4), at 30 days (n = 2) or six months (n = 2) after arrest (Table 1). Overall, patients treated with TTM at 33 °C showed a similar probability of mortality (OR 1.01 [95% CIs 0.90–1.14] *p* = 0.87) than those treated at 36°C (Figure 3).

**Figure 4:**
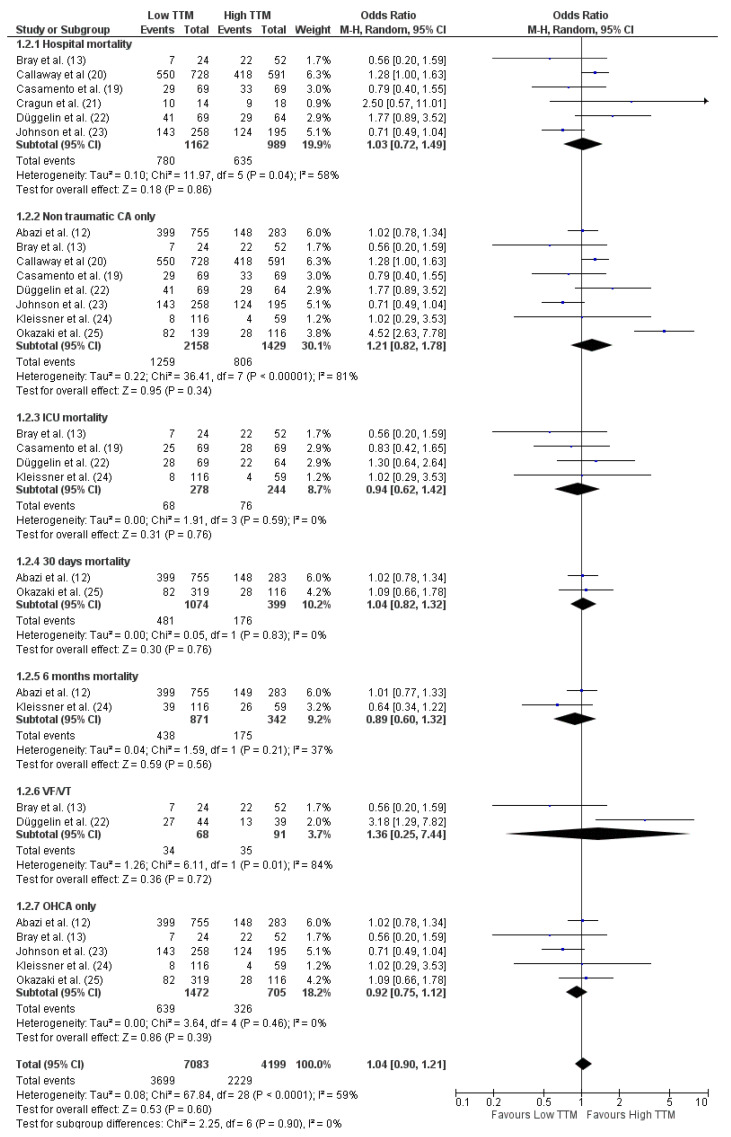
Forest plot for mortality in all patients at different time-points. Low TTM = TTM at 33 °C; High TTM = TTM at 36 °C. The size of the squares for the risk ratio reflects the weight of the trial in the pooled analysis. The horizontal bars represent 95% confidence intervals (CIs). OHCA = out-of-hospital cardiac arrest; VF/VT = ventricular fibrillation/ventricular tachycardia [12,13,19,20,21,22,23,24,25].

Subgroup analysis analyzing survival at different time-points also showed no differences between the two groups; however, high heterogeneity was observed in some of the analyses. No differences in mortality were observed when mortality was analyzed only in patients with an initial shockable rhythm (Figure 4).

## 4. Discussion

Many centers have adopted a target temperature of 36 °C for the management of cardiac arrest patients suffering from HIBI [26,27]; overall, the mean temperature during the cooling phase has also progressively risen during the first 24 h of treatment after the publication of the TTM study [28]. This strategy is consistent with the results of this RCT [10], which reported a similar mortality and UO rate in the TTM at 33 °C and TTM at 36 °C groups, even in predefined subgroups (i.e., age, gender, time to return of spontaneous circulation, initial rhythm, presence of shock). Moreover, the larger inclusion rate of eligible patients in this study, when compared to the small and selected cohorts included in previous ones [5,6], resulted in a broader generalizability of these findings to heterogeneous cardiac arrest populations. Despite the decision to move to TTM at 36 °C as the “new standard of care” being initially supported by the neutral effect of a lower target temperature on clinically relevant outcomes, the lower number of complications than TTM at 33 °C and the ease to maintain this target (i.e., similar to admission temperature of cardiac arrest patients and close to normothermia), no *post hoc* analyses from the TTM database showed any clear advantage of TTM at 36 °C on TTM at 33 °C, including the occurrence of infections, acute kidney injury or seizures [29,30,31]. TTM at 33 °C was associated with more hyperglycemia, more frequent decreased heart rate, elevated lactate levels and need for vasopressors and a longer time to awakening when compared with TTM at 36 °C [32,33,34], although none of these issues influenced outcome differences between groups. Although the warning not to abandon strict temperature control in favor of normothermia or avoidance of fever as this approach was not tested in the TTM study [35], some studies reported an increased frequency of fever in cardiac arrest patients since 2013 [13,28], which was not observed in the original RCT and might be associated with an increased risk of poor outcome.

Because of this discrepancy between a pragmatic RCT and “real life “data, we performed this systematic research and meta-analysis, aiming to summarize how the translation of the TTM-study results in clinical practice may have affected patients’ outcome. Accordingly, we observed a similar mortality rate between the two TTM strategies, but a lower probability of UO when TTM at 33 °C was applied. We based this statement on the analysis of UO as CPC 3–5, because one study reported neurological function using the ability of the patient to follow commands, which is clearly too limited to assess adequate recovery. Do these findings question the validity of the TTM study? Certainly not. This RCT had a high-quality methodological design (i.e., large cohort; long-term outcome; blinded outcome assessor; reduced random effect) and adequately compared the two TTM strategies. On the opposite, this meta-analysis was based only on retrospective studies (i.e., very low quality of evidence), including patients with imbalanced characteristics at the baseline between TTM at 33 or 36 °C, with different quality of TTM (i.e., different protocols, cooling devices, temperature monitoring) and relatively short-term outcome report (i.e., most at hospital discharge). Moreover, the definition of UO was not standardized among studies and potentially not adequately assessed as in a prospective or randomized trial.

Nevertheless, our results can at least cause some debate on how target temperature should be decided in the modern management of cardiac arrest patients. First, the application of the “TTM protocol” (i.e., induction of cooling; maintenance of 24 h; use of sedation during the cooling phase; relatively slow rewarming, avoidance of fever for 72 h; specific protocol for neuro-prognostication) might have been inadequately translated into clinical practice and resulted in more patients with early fever and poor temperature control. In one study including only OHCA with a shockable rhythm, patients treated with TTM at 36 °C spent less time outside the target temperature, with most of them having a greater probability to have at least one episode >38 °C [13]; this was associated with a trend towards an increased mortality (42% vs. 29%) and occurrence of UO at hospital discharge (44% vs. 29%) when compared to patients treated with TTM at 33 °C, although the results were not statistically significant. Of course, the importance of an adequate temperature control is important for both target temperatures; in one of the selected studies, TTM at 36 °C was associated with a higher protocol adherence (i.e., body temperature was within ±1 °C around the target temperature of 33 °C or 36 °C) and had a numerically lower mortality (34% vs. 41%) and UO rate (55% vs. 65%) than the TTM 33 °C group [20]. A second important issue is the design of the studies included in this meta-analysis; as most of them had a “before” (i.e., TTM at 33 °C, until 2013) vs. “after” (i.e., TTM at 36 °C, after 2013) comparison, the improvement of general care of these critically ill patients over time should have benefit the TTM 36 °C group, while our results showed the opposite. As such, the misinterpretation of the TTM trial results have probably induced many clinicians to change the quality of their practice (i.e., less accurate temperature control in cardiac arrest patients), which might also potentially result in a less accurate general surveillance of other important parameters, such as hemodynamics, gas exchanges and the occurrence of organ failure. If these effects do not influence survival, neurological outcome can potentially be altered; indeed, brain is the most sensitive organ to temperature changes after the anoxic injury [4] and a RCT showed that TTM at 33 °C improved neurological function, but not survival, in OHCA with an initial non-shockable rhythm when compared to normothermia [7]. A third important issue is related to the consistent effects of TTM at 33 °C on UO when compared to 36 °C in all selected studies, with the exception of one where TTM at 33 °C was provided with a poor adherence of temperature protocol [22] and a second including only traumatic and post-operative cardiac arrest [21], which is generally excluded from large RCTs.

Unfortunately, our results do not help to individualize target temperature to patients’ characteristics. After excluding patients with initial severe cerebral edema and early highly malignant patterns on electroencephalography (EEG), Callaway et al. [20] observed that TTM at 33 °C was associated with a lower mortality at hospital discharge in “severe” patients (i.e., severe coma or cardiovascular failure) when compared to TTM at 36 °C, while the opposite was observed among patients with mild to moderate coma without shock. In another study, Okazaki et al. [25] reported a lower mortality in patients with initial lactate levels exceeding 12 mmol/L when treated with TTM at 33 compared to 36 °C. Although the need for a “tailored” therapy is needed, none of the available data could identify a subgroup of patients who will definitely benefit from one of the two TTM strategies.

## 5. Conclusions

TTM is a relatively safe and effective strategy to improve the neurological outcome of cardiac arrest patients who remain comatose after hospital admission. Many questions on its effectiveness and implementation remain still unanswered; the translation of results from RCTs in clinical practice should be carefully evaluated as misinterpretation of such results might influence patients’ outcome. The use of TTM protocols aiming at avoiding poor temperature control is mandatory. However, the optimal approach to individualize target temperature based on patients’ characteristics remains still elusive. As for all newly commercialized drugs, epidemiological studies and surveillance programs with an adequate follow-up on large databases are necessary to understand how results from RCTs translate into medical practice.

## Figures and Tables

**Table 1:** Summary of the selected studies comparing target temperature management at 33 and 36 °C. For mortality and neurological outcome assessment, “ICU” and “Hospital” refer to ICU discharge and hospital discharge.

First Author, Year [Ref]	Type of Study	Number	OHCA	IHCA	VF/VT	Mortality Assessment	Definition UO	UO Assessment
Abazi, 2019 [12]	R	1038	YES	NO	673	30-day 6-month	NR	NR
Bray, 2017 [13]	R	76	YES	NO	76	ICU Hospital	CPC 3–5	Hospital
Callaway, 2020 [20]	R	1319	YES	YES	369	Hospital	CPC 4–5 CPC 3–5	Hospital
Casamento, 2016 [19]	R	138	YES	NR	86	ICU Hospital	NR	NR
Cragun, 2018 [21]	R	32	YES *	YES **	NR	Hospital	Unable to follow commands	Hospital
Düggelin, 2020 [22]	R	133	YES	YES	83	ICU Hospital	CPC 3–5	Hospital
Johnson, 2020 [23]	R	453	YES	NO	173	Hospital	CPC 3–5	Hospital
Kleissner, 2019 [24]	R	1710	YES	NO	866	Hospital	CPC 3–5	Hospital
Okazaki, 2019 [25]	R	435	YES	NO	235	30-day	CPC 3–5	30-day

OHCA = out of-hospital cardiac arrest; IHCA = in-hospital cardiac arrest; R = retrospective; ICU = intensive care unit; VF/VT = ventricular fibrillation/ventricular tachycardia; NR = not reported; UO = unfavorable neurological outcome; CPC = Cerebral Performance Category. * = trauma; ** = post-operative.
